# The Representation of Prediction Error in Auditory Cortex

**DOI:** 10.1371/journal.pcbi.1005058

**Published:** 2016-08-04

**Authors:** Jonathan Rubin, Nachum Ulanovsky, Israel Nelken, Naftali Tishby

**Affiliations:** 1 Edmond and Lily Safra Center for Brain Sciences, Hebrew University, Jerusalem, Israel; 2 Department of Neurobiology, Weizmann Institute of Science, Rehovot, Israel; 3 Department of Neurobiology, Institute of Life Sciences, Hebrew University, Jerusalem, Israel; 4 The Benin School of Computer Science and Engineering, Hebrew University, Jerusalem, Israel; University of California at Berkeley, UNITED STATES

## Abstract

To survive, organisms must extract information from the past that is relevant for their future. How this process is expressed at the neural level remains unclear. We address this problem by developing a novel approach from first principles. We show here how to generate low-complexity representations of the past that produce optimal predictions of future events. We then illustrate this framework by studying the coding of ‘oddball’ sequences in auditory cortex. We find that for many neurons in primary auditory cortex, trial-by-trial fluctuations of neuronal responses correlate with the theoretical prediction error calculated from the short-term past of the stimulation sequence, under constraints on the complexity of the representation of this past sequence. In some neurons, the effect of prediction error accounted for more than 50% of response variability. Reliable predictions often depended on a representation of the sequence of the last ten or more stimuli, although the representation kept only few details of that sequence.

## Author Summary

A crucial aspect of all life is the ability to use past events in order to guide future behavior. To do that, creatures need the ability to predict future events. Indeed, predictability has been shown to affect neuronal responses in many animals and under many conditions. Clearly, the quality of predictions should depend on the amount and detail of the past information used to generate them. Here, by using a basic principle from information theory, we show how to derive explicitly the tradeoff between quality of prediction and complexity of the representation of past information. We then apply these ideas to a concrete case–neuronal responses recorded in auditory cortex during the presentation of oddball sequences, consisting of two tones with varying probabilities. We show that the neuronal responses fit quantitatively the prediction errors of optimal predictors derived from our theory, and use that result in order to deduce the properties of the representations of the past in the auditory system. We conclude that these memory representations have surprisingly long duration (10 stimuli back or more), but keep relatively little detail about this past. Our theory can be applied widely to other sensory systems.

## Introduction

Organisms often operate in unknown and uncertain environments. Therefore, extracting aspects of past observations, which are maximally predictive of the relevant future, is essential for survival. It has been suggested that the sensory cortex evolved to extract the statistical regularities of the world [1]. Adaptation of the nervous system to the statistical structure of the input is reflected in studies of neuronal responses to natural stimuli. For example, in the auditory system, auditory nerve fibers–part of the auditory periphery–have been shown to achieve high coding efficiency by implementing a “tuned” nonlinear filter that selectively amplifies the anticipated signal [2]. Similarly, in the visual system, Laughlin [3] showed that the contrast-response function of interneurons in the fly's compound eye approximates the cumulative probability distribution of contrast levels in natural scenes.

The central auditory system shows sensitivity to stimulus statistics as well. Event-related potentials recorded in humans show sensitivity to deviant stimuli. This sensitivity may occur rather early, at the mid-latency potentials range [4], and has been intensively studied in the context of the mismatch negativity (MMN), peaking about 150 ms after the point of deviance [5]. Similar sensitivity occurs also in the responses of single neurons in auditory cortex: using oddball sequences composed of two frequencies with different probabilities, Ulanovsky et al. [6] found that neurons in cat auditory cortex responded more strongly to a given tone frequency when it was rare than when it was common. This sensitivity, named stimulus-specific adaptation (SSA) by Ulanovsky et al. [6], has been by now shown in multiple mammalian species and even in birds (See [7] for review). SSA may be linked to deviance detection in the time frame of the mid-latency potentials rather than to MMN (Grimm, Escera and Nelken 2015). Thus, we hypothesize that neurons in the auditory system encode some notion of a prediction error.

The coding of prediction error is considered central to learning [8], memory formation [9], and decision-making [10]. Prediction errors are also known to be related to efficient coding where only the unexpected at one stage of processing should be transmitted to the next stage [11].

This paper has two goals. The first is to present a theory of prediction error from first principles. For an organism that operates in a statistically stationary world (as is generally the case in laboratory experiments), prediction quality is limited by the statistical structure of the data that the organism collects from the past. The theory depends only on this statistical structure, and not on any assumptions about specific brain mechanisms. We assume that the brain forms a *reduced representation* of the past, which serves to generate predictions of future events. We use information theory to quantify both the *complexity* of these reduced representations and the *predictive information* they carry with respect to future events [12]. The term complexity refers here to the rate of information (specifically, the mutual information in bits/s or, equivalently but more conveniently here, in bits/stimulus) that the representation carries about the past. Predictive information refers to the rate of information (again, mutual information in bits/s or bits/stimulus) that the reduced representation carries about future events. Both terms are defined precisely below. Extraction of the predictive aspects of the past stimuli can be formalized as an optimization problem: minimize the complexity of the reduced representation of the past while preserving a predefined level of predictive information. A reduced representation provides a predictive probability for every event–this is the probability assigned to the event just before it actually occurred, given the reduced representation of the past. Our theory uses these predictive probabilities to calculate prediction errors–events with low predictive probability generate large prediction error, while events with a high predictive probability generate small prediction errors.

The constrained optimization problem we use to calculate the reduced representations is a special instance of the *Information Bottleneck* (IB) principle [13]. The IB principle applies to any two random variables X and Y with a known joint distribution. Like the special case described here in details, the IB principle provides a way of finding reduced representations (as defined later in the paper) of X that are maximally informative about Y. Here we apply it to the past of the stimulation sequence (X) and to its future (Y). Much of the detailed discussion below can be considered as a primer for the use of the IB principle.

The second goal of the paper is to demonstrate the usefulness of the theory by applying it to the neuronal responses evoked by random tone sequences consisting of two frequencies with varying probabilities (‘oddball’ sequences). We show that prediction errors calculated by the theory correlate well with neuronal responses. Most importantly, we use the theory to extract parameters of the reduced representations that underlie these responses: we show that these reduced representations have a long duration (typically N ≥ 10 preceding stimuli) but keep only coarse details about the sequence that was presented (i.e., the reduced representations have low complexity). These results show how to establish properties of the neural code rigorously from first principles.

## Results

**Fig 1:**
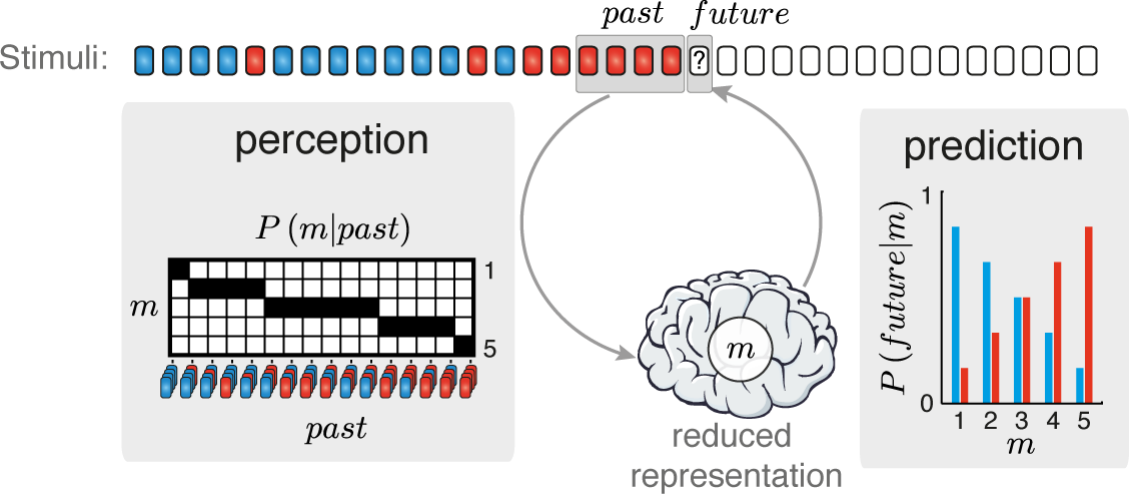
Information flow between the organism and the environment. The environment consists of a stationary random process. Here the environment produces a Bernoulli sequence of two stimuli. The organism perceives the sequence, summarizing the recent past by a state of a reduced representation (*m*), then uses *m* to produce a prediction for the next stimulus in the sequence. Perception is characterized by *P(m|past)*, mapping a sequence of *past* observations (*N* = 4 in this illustration) into states *m* of the reduced representation. In this example, the reduced representation consists of the number of red stimuli among the last *N* stimuli. Prediction is characterized by *P(future|m)*, which assigns to each state *m* a set of subjective expectations for the next (*future*) stimulus. As the number of red stimuli in the past increases, the probability assigned to a red stimulus increases and that assigned to a blue stimulus decreases. The numbers shown here correspond to the posterior probabilities for the corresponding stimulus given a uniform prior on the probability of a red stimulus.

We treat the process of perception and prediction within an information theoretical framework, where information flows between the environment and the organism (**Fig 1**). In the following, we describe the general framework and at the same time apply it to the special case of random two-tone sequences that will be used later to show that the theory can be applied exactly to (admittedly simple) realistic scenarios.

The starting point of the theory is a stationary ‘world’–in practice a sequence of stimuli, with a known probability distribution. To simplify the theoretical treatment, we assume the stimuli occur at a fixed rate and have a fixed duration. For the special case of two-tone sequences, we assume that the stimuli are single tones of one of two possible frequencies. For each stimulus presentation, one or the other of the two frequencies are selected with probabilities *p* and 1 − *p* which are fixed along the sequence. We also assume that choices of the frequency in subsequent trials are independent. To avoid confusion later in the paper, the terms ‘frequency’ and ‘probability’ will always be used in the same way–frequency refers to tone frequency, probability to its probability of occurrence. Such sequences are commonly used to study MMN in humans as well as SSA in animal models [14]. Typically, in these sequences the tone duration is relatively short–tens or a couple of hundreds of ms–whereas the inter-stimulus interval is much longer, e.g. 300 ms or 730 ms or 1200 ms. In the data analyzed later in the paper, tone duration was 230 ms and the inter-stimulus interval was 730 ms.

We conceptualize the organism as a prediction machine (**Fig 1**)–it has to predict the next stimulus as accurately as possible. In particular, we assume that the organism forms a reduced representation of the past stimuli (in some predefined time window, to be specified later), which it uses to generate predictions of future events. The reduced representation is a (potentially probabilistic) function of the recent history of the stimulation sequence.

We treat the observed sequence of stimuli, its reduced representation, and the next stimulus, as three random variables denoted respectively by *past*, *m*, and *future* (**Fig 1**). The reduced representation of the past is specified by a conditional probability distribution *P(m|past)*, mapping a sequence of *N* past stimuli into states of *m* (*N* will be discussed further below). This is the sense in which the reduced representation is a function of the past. Predictions are carried by another conditional probability distribution *P(future|m)* (the predictive probability distribution) that assigns a set of expectations for future events to each state of *m*, and is therefore a probabilistic function of the reduced representation. Importantly, all uses of the term ‘representation’ in this paper refer to the notion as defined here–a random variable *m*, which is a function, potentially probabilistic, of the past, with its associated predictive probability distribution. A reduced representation is therefore fully characterized by the two associated conditional probability distributions, *P*(*m*|*past*) and *P*(*future*|*m*). As usual in probability theory, by abuse of notation we will use *m* to denote both the random variable (specified by the conditional probability *P*(*m*|*past*)) and the specific values it can take (the ‘states’ of the reduced representation). The meaning should be clear from the context. We will derive the reduced representations (and corresponding predictive probability distributions) from the IB principle as described later.

**Fig 2:**
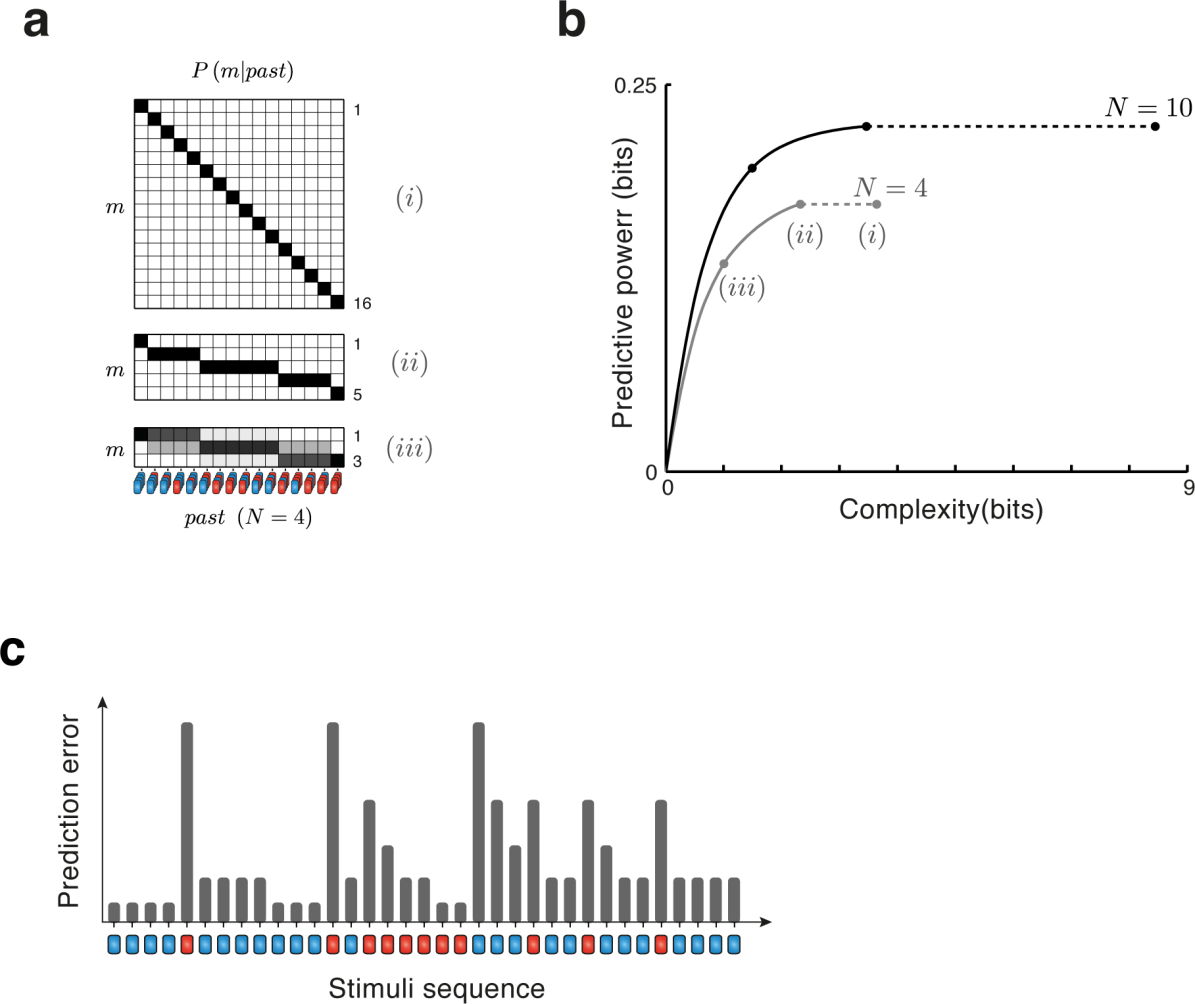
Reduced representations through the Information Bottleneck method. (**a**) Illustration of different reduced representations of past sequences in the oddball paradigm (for *N* = 4 stimuli). Reduced representations are depicted by the conditional probability distribution *P(m|past)* that maps sequences of observations into states *m*. (*i*) The topmost representation maps each possible sequence of *N* = 4 stimuli to unique state *m*, resulting in 16 states. (*ii*) The middle representation is a reduced version, in which sequences with the same number of occurrences of each tone are grouped together resulting in 5 different states. This is the minimal sufficient statistic, and is also the representation illustrated in Fig 1. (*iii*) The bottom representation with only 3 states results from further constraining the complexity (mutual information between the reduced representation and the past) to 1 bit, and is the optimal representation (with highest predictive power) with that complexity. For this representation, the mapping for past to the state of the representation is probabilistic. (**b**) The tradeoff between predictive power and complexity of reduced representations in the oddball paradigm for two durations of the past (*N* = 4 stimuli in gray; *N* = 10 stimuli in black). Each point along the solid curves shows the complexity (abscissa) and predictive power (ordinate) of one unique solution of the tradeoff, with maximal predictive power for the given complexity and, equivalently, minimal complexity for the given predictive power. The rightmost points correspond to the complexity and predictive power of the full representations that assign a unique state *m* to each and every possible sequence of *N* stimuli. Dashed lines connect these values with the points corresponding to the representations based on the minimal sufficient statistic (number of red stimuli). Using the minimal sufficient statistic produces representations that are less complex but provide the same predictive power as the full representations. Further constraints on the complexity result in representations that have lower predictive power. The complexity and predictive power of representation (iii) are shown explicitly on the N = 4 curve: the complexity of this representation is lower than that of the sufficient statistic, and in consequence its predictive power is lower as well. (**c**) Prediction errors along an oddball sequence calculated using the transformation illustrated in panel **a** ((i) and (ii), which produce the same prediction errors). In this example, for each stimulus, the preceding four stimuli determine a unique state *m* of the reduced representation; prediction error is calculated from the predictive distribution using that state *m*. Bar heights represent the prediction error associated with each stimulus.

As a concrete application of the theory to oddball sequences, assume that the temporal window extending to the past is of length *N = 4* stimuli (**Fig 1**) and that we want to predict the frequency of the next tone. The oddball sequences are assumed to be generated in the following way: before the beginning of each sequence, the experimenter selects a probability *p*. Then, for each tone presentation, the experimenter selects to use tone ‘A’ (blue in Fig 1) with probability *1-p* and tone ‘B’ (red in Fig 1) with probability *p*. Importantly, while the probability of the two tones is selected *a-priori* by the experimenter, it is unknown to the subject of the experiment, and therefore the past and future of the stimulation sequence are not independent of each other. Instead, the relative abundance of 'A' and 'B' tones in the near past carries information about the probability of the next tone frequency. In this example, the past random variable is simply the list of the last four tone frequencies (‘A’ or ‘B’) in the order in which they occurred, and the future variable is the next tone in the sequence. We next need to specify the reduced representation, *m*. One obvious choice consists of setting *m* to be the full past, that is, the list of the last four tone frequencies in the order in which they occurred. In that case, the reduced representation is not really reduced, *P(m|past) = 1* for the state of the representation that corresponds to the past that actually occurred and *P(m|past) = 0* otherwise (**Fig 2A** case (i)).

However, it is clear that this representation is too detailed. In order to predict the next tone as accurately as possible from an oddball sequence, where the stimuli are independent and identically distributed (i.i.d), it is enough to know the number of B tones that occurred among the last 4 tones. This number is a *minimal sufficient statistic* in the case of the oddball sequences: given this number, no additional information about the past (e.g. the order in which these four tones were presented) can improve the prediction of future tone frequencies. If we choose *m* to be the number of B tones among the last four tones, we have a reduced representation that can be in 5 different states, corresponding to 0 B tones, 1 B tone and so on up to 4 B tones. Some of the conditional probabilities that define *m* are for example *P(m = 0|AAAA) = 1*, *P(m = 3|ABAA) = 0*, *P(m = 2|ABAB) = 1*. The matrix that defines this reduced representation is shown in Figs 1 and 2A case (ii).

A third possible reduced representation is shown in Fig 2A (iii) ('case (iii)' below). While cases (i) and (ii) are deterministic, case (iii) is a probabilistic (‘soft’) assignment of the past into 3 classes, which can be roughly characterized as ‘a lot of A tones’, ‘about the same number of A and B tones’, and ‘a lot of B tones’. The assignment is soft in the sense that it allows each past to be assigned with some non-zero probability to each of the three possible states of the reduced representation. Thus, the past ABAA is assigned with high probability to the class ‘a lot of A tones’, but it has some probability to be assigned to the class ‘about the same number of A and B tones’. Similarly, the past AABB is assigned with high probability to the class ‘about the same number of A and B tones’, but has some probability to be assigned to either of the other two classes.

While such probabilistic reduced representations may seem somewhat contrived at this point, we will see later that they actually occur naturally as part of the theory, as optimal solutions to the problem of simplifying (in a sense to be made precise below) the minimal sufficient statistic. Such simplifications may be required because of imperfect or constrained sensory capacity–for example, at the time that the presentation of the last tone ends and the state *m* is established, the identity of the tones has been already forgotten to some degree. As a result, there is some uncertainty about the past sequence, reflected in the soft character of such reduced representation.

To complete the specification of the reduced representations for cases (i)-(iii), we need, in addition to the mapping *P*(*m*|*past*), to specify the predictive probability distributions *P*(*future*|*m*) for each case. In all three cases, *P*(*future*|*m*) is a specification of the probabilities for the A and B tones to occur, and these sum to 1. It can therefore be specified by a single number, the probability of the B tone given the state of the reduced representation. In case (i), a naïve choice for this probability would be *P(B|m) = #(B tones)/4*. This choice turns out not to be optimal (the optimal choice depends on the *a-priori* distribution of *p*). For case (ii), the similarly naïve choice would be *P(B|m) = m/4*. For case (iii), we have to specify three probabilities, one for each class of the reduced representation. Clearly, for the class corresponding to ‘about the same number of A and B tones’, the predictive probability for a B tone should be 0.5, whereas it is smaller for ‘a lot of A tones’ and larger for ‘a lot of B tones’. The exact values will be derived later.

We go back to the general theory. Given that there are many possible reduced representations (for example, we described at least three different ones in Fig 2A), we have to specify selection rules–which reduced representations are good and which are not. This is really the heart of the theory. The IB principle postulates that the set of good reduced representations is formed of the solutions to a tradeoff between the quality of the predictions that the reduced representation provides about the future, and how complex the reduced representation is. We now define precisely what we mean by these terms.

To quantify the quality of the predictions derived from a given reduced representation we first compute prediction errors. Fig 2C illustrates the derivation of the prediction errors. To calculate the prediction error associated with the occurrence of a given stimulus, we consider the N stimuli that just preceded it as the past, and the given stimulus as the upcoming, ‘future’, stimulus. In Fig 2C, for example, when considering a past whose duration is N = 4 stimuli, the first B tone (red, 5^th^ tone in the sequence) has a past that is composed four successive A stimuli (in blue). Once these four stimuli occurred, we can use the reduced representation in order to predict what would be the upcoming (future) stimulus–the red tone in this case. To do that, we use the probability distribution *P*(*m*|*AAAA*) (part of the specification of the reduced representation) to select a specific state for *m*. For the example of Fig 2C, we use case (ii), so that the state *m* is determined uniquely as *m* = 1 (see Fig 2A). We now determine the probability of the stimulus that actually occurred, the B tone, which is *P*(*B*|*m* = 1) (again, part of the specification of the reduced representation). As discussed above, this probability should be small (close to 0) for any reasonable reduced representation.

We can repeat the same process for all locations along the sequence, assigning a predictive probability for each stimulus–the probability with which that specific stimulus is expected, given the state *m* of the reduced representation, determined from the immediate past of the stimulation sequence. For example, in Fig 2C, the next 3 stimuli are A tones (blue), their immediate past is AAAB, AABA and AAAB respectively, all three of which are mapped into state m = 2 (see Fig 2A, case(ii)). The probability for observing A when m = 2 should be relatively large (although smaller than the probability for observing A when m = 1).

We define the prediction error by −log_2_(p), where *p* is the probability with which the future event is expected given the state *m* of the reduced representation that corresponds to the immediate past, as described in the preceding paragraphs (**Fig 2C**; see Methods for details). In the case of the oddball sequences, *p* will be simply *p(B|m)* if tone B occurred, and *1-p(B|m)* if tone A occurred. This choice conforms with the intuition that a stimulus that had small predictive probability is more surprising than a stimulus with large predictive probability. Indeed, for probabilities close to 0, -log(p) is large, while for probabilities close to 1, -log(p) is close to 0 (in both cases, it is positive, since p<1). In Fig 2C, for example, the prediction error at the 5^th^ position is large, since the past had 4 A tones but the future was a B tone, while the next 3 prediction errors are smaller, since at those positions that past had 3 A’s and 1 B tones, and the future was an A tone. As importantly, this is essentially the only consistent choice for prediction error if we require it to have a small number of natural properties ([15], an excellent intuitive discussion can be found in [16]).

The prediction error fluctuates on a trial-by-trial basis, so it cannot be used ‘as is’ to quantify the quality of the predictions based on the reduced representation. Furthermore, even when the sequence of stimuli is given, the state *m* can still be a random variable; therefore, the prediction error is itself a random variable. Thus, the relevant quantifier is the expected value of the prediction error, which is well-defined for a stationary process. Even the expected prediction error itself is technically somewhat inconvenient to use. Instead, we use a simple transformation of the expected prediction error. In the Methods section, we demonstrate that the prediction error is equal to the difference *H*(*future*) − *I*(*m*; *future*), where *H*(*future*) is the entropy of the upcoming stimulus (which doesn’t depend on the reduced representation) and *I*(*m*; *future*) is the mutual information between the reduced representation and the upcoming stimulus. In consequence, the expected prediction error and the mutual information *I*(*m*; *future*) are negatively related to each other. While the expected prediction error is the quantity of interest, the mutual information is easier to work with. Therefore, we define the *predictive power* of a reduced representation *m* as the mutual information between the reduced representation and the upcoming stimulus, *I(m;future)*. The larger it is, the better are the predictions derived from the reduced representation.

We turn now to the definition of the *complexity* of the reduced representation. The past may include a substantial amount of details that are irrelevant for predicting the future. These details can be ignored in the reduced representation without affecting prediction quality. Our definition of complexity relates to this relationship between the reduced representation and the past, and it encodes the amount of details that the reduced representation extracts from the past sequence in order to make predictions about the future. This notion is quantified by the mutual information *I(past;m)*. A reduced representation of lower complexity keeps less detail about the past than a reduced representation of higher complexity.

The use of mutual information to measure predictive power and complexity provides the theory with absolute scales. The complexity cannot be larger than the entropy of the past, so that a reduced representation whose complexity is equal to that of the past is equivalent to storing the full details of the past stimulation sequence. Similarly, the predictive power cannot exceed the entropy of the future, but in general, it has a tighter bound: even the best representation of the past (equivalent to storing the full past) cannot in general achieve full predictability of the future. In fact, the predictive power is bounded by *I(past;future)*, which is generally much smaller than the entropy of the future.

These definitions link the theory in a natural way with the notions of sufficient statistics and minimal sufficient statistics [13, 17]. A reduced representation that achieves the upper bound on predictive power (the mutual information between past and future) is a ‘sufficient statistic’ in the sense of classical statistical theory and a sufficient statistic of minimal complexity (by our definitions, and the Data Processing Inequality) is a ‘minimal sufficient statistic’ in the same sense ([18] pp. 115–119). We are however interested also in reduced representations that are simpler than the minimal sufficient statistic. Such reduced representations cannot achieve the full predictive power, but as we will show below, substantial reductions in complexity can result in rather minimal losses of predictive power.

In the case of the oddball sequences, we can explicitly calculate the predictive power and complexity of the reduced representations. For **Fig 2A** case (i), complexity is 3.64 bits and predictive power is 0.173 bits. For case (ii), complexity is 2.32 bits and predictive power is 0.173 bits. Note that case (i) has the same predictive power as case (ii), but a higher complexity. Thus, case (i) is in a sense suboptimal. In fact, case (ii) is the simplest possible among all reduced representations that achieve the maximal predictive power, a reflection of the fact that it is the minimal sufficient statistic for estimating the probability of tone B ([18] pp. 92–95). For case (iii) (with optimally specified probabilities, as described later) the complexity is 1 bit and predictive power is 0.134 bits. While case (iii) has lower predictive power than case (ii), the decrease in predictive power (by a factor of 0.77) is much smaller than the decrease in complexity (by a factor of 0.43). Case (iii) is in fact the reduced representation with minimal complexity among all those whose predictive power is not smaller than 0.134 bits.

Going back to the general theory, the IB principle posits that in general, among all reduced representations that achieve a certain level of predictive power (such as e.g. cases (i) and (ii) above), the one that has the lowest complexity is the best. We can always achieve maximal predictive power by setting *m* to be the full past (as in case (i) above), and try to simplify it as we did when going from case (i) to case (ii). However, simple sufficient statistics (simpler than storing the entire past) are available only for a specific class of distributions (exponential families; [18] pp. 102–110), and even then may be too complex (e.g. requiring a sensory resolution that is too high). Thus, we want to consider reduced representations that are less complex than the sufficient statistics, paying the price of potentially reducing the predictive power. In fact, we consider the reduced representation to be a ‘bottleneck’ between perception (coding of the past) and prediction. Case (iii) suggests that by accepting minor reductions in the predictive power, we can produce in many cases reduced representations of the oddball sequences that are considerably simpler than even the minimal sufficient statistic.

We want therefore simple reduced representations that are have high predictive power, or alternatively reduced representations with high predictive power that are simple. The two requirements are in opposition to each other–the simpler the reduced representation is, the lower the predictive power we can expect it to have. This is the essential tradeoff posited by the IB principle. A good reduced representation is one that has maximal predictive power given its complexity, or alternatively that is the simplest possible given its predictive power.

Technically, tradeoffs of this kind are solved by constrained maximization. We impose a constraint on the predictive power, requiring it to have a value *I*_*pred*_ within its allowed range, and then search for the reduced representation that has that predictive power but minimal complexity. The search is performed over all reduced representations–that is, conditional probability functions *P*(*m*|*past*) and *P*(*future*|*m*)–which have the desired level of predictive power. However, given *P*(*past*|*future*) (which is assumed to be known *a-priori*), *P*(*future*|*m*) can be computed from *P*(*m*|*past*). Thus, it is enough to optimize over all *P*(*m*|*past*) that fulfill the constraint.

Formally, given a statistical characterization of the stationary stimulation process *P(past*,*future)*, we look for a reduced representation with minimal complexity that has the imposed level of predictive power (s.t. stands for ‘such that’):

$$\underset{P(m|\mathrm{past})}{\min}I\;(past;m)\;s.t.\;I(m;future)=I_{pred}.$$

Alternatively, we can restate the optimization principle by considering its dual problem: find a reduced representation of the past with some desired level of complexity that maximizes its predictive power (and thus minimizes the expected prediction error).

$$\underset{P(m|\mathrm{past})}{\max}I\;(m;future)\;s.t.\;I(past;m)=I_{complex}$$

These two dual problems are the mathematical expression of the IB principle [13]. A solution to these dual problems consists of the two conditional distributions: *P(m|past)* and *P(future|m)* that characterize how the state of the reduced representation is determined from the past and how predictions are carried out given the state of the reduced representation. There is a family of such solutions, determined by the values of the constraints *I*_*pred*_ or *I*_*complex*_. The solutions can be found by applying the Information Bottleneck algorithm [13], an iterative algorithm that solves the constrained optimization problem for all allowed values of the constraints, given the joint probability distribution of past and future. For details, see Methods. A given solution to one problem will be also a solution to the other one: it will have the maximal predictive power among all models that have the same complexity, and will have the minimal complexity among all models that have the same predictive power.

### Information Bottleneck Analysis of the Oddball Paradigm

**Fig 3:**
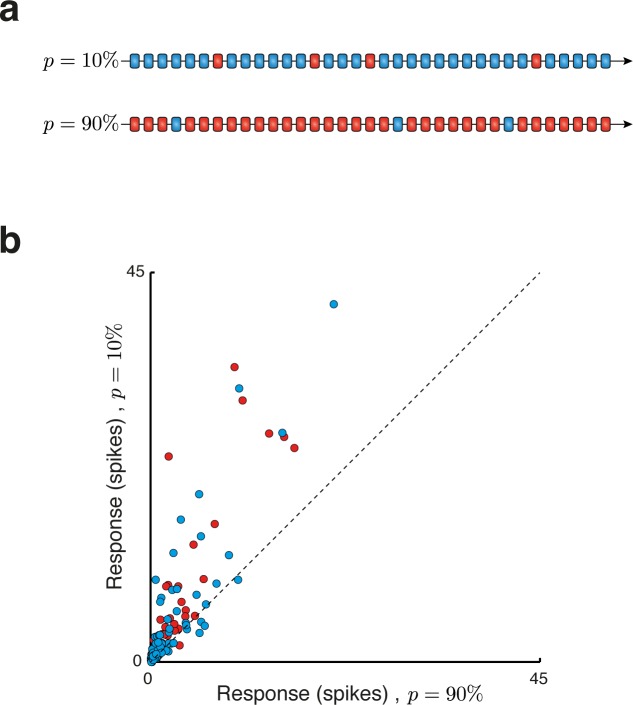
Neural responses in the oddball paradigm. (**a**) Two examples of oddball stimuli blocks, one with *p* = 10% and the other with *p* = 90%. Different colors (blue and red) represent the two stimuli (‘low’ and ‘high’ tones of the oddball sequences). (**b**) Single-neuron responses in cat primary auditory cortex (A1) to auditory oddball stimuli. Each point shows the average spike counts of one neuron to the same stimulus (either low-tone or high-tone, represented by different colors) when presented in the *p* = 10% block versus the same physical stimulus in the *p* = 90% block. Note that most dots fall above the diagonal, indicating stronger response to rare stimuli.

In order to demonstrate the relevance of our approach to neuronal processing, we studied responses of single neurons in primary auditory cortex (A1) to oddball sequences. Oddball sequences are generated by selecting two stimuli (two pure tones in our case) and then forming a sequence composed of these two stimuli in which one of the two is common and the other is rare (**Fig 3A**). Usually, the overall number of times each of the stimuli occur in the sequence is fixed. We will however test here a slightly different statistical model, in which the probability of each of the tones is fixed. We will furthermore assume that successive tones are selected independently of each other with a given probability (‘Bernoulli sequences’). This assumption, which is a very good approximation to the experimental setting when the number of tones in the sequence is large, makes the application of the theory particularly transparent.

Neurons in auditory cortex are sensitive to stimulus probability in such sequences [6, 19–23]. The majority of neurons in cat auditory cortex produced a larger average response to the same tone frequency when it was rare than when it was common (**Fig 3B**). These responses often had substantial sustained components that could outlast stimulus duration (e.g. [24]), resulting in large spike counts within the counting window we used (0–330 ms after stimulus onset, for stimuli whose total duration was 230 ms, same as in [6, 20]). We interpret these responses as encoding a prediction error: in the oddball sequences, the probability of the next stimulus to be either of the two frequencies is roughly given by the probability with which it occurred in the past. The prediction error, −log_2_(*p*), for the tone when rare is therefore larger than for the same tone when common. Thus, responses of neurons in auditory cortex seem to fit qualitatively the notion of prediction error as defined here.

Using the theory presented above, we aimed to quantify this intuition rigorously. We start by describing in substantial detail the family of optimal reduced representations in the case of the oddball sequences, and then we show how we used these representations to extract information about the memory duration and complexity of the reduced representation underlying the dependence of neuronal responses in auditory cortex on tone probability.

In the experiments, sequences with a number of different probability values have been used: each stimulus was used both as common (with probabilities of 90% and 70%) and as rare (with probabilities of 10% and 30%), as well as in equiprobable sequences. Formally, the stimulus sequence can be modeled as a Bernoulli process, where one stimulus is drawn with probability *p* (0<*p*<1) and the other with probability 1-*p*. We assume that the parameter *p* is drawn from a uniform distribution prior to generating each stimulus block. This assumption is not crucial–it changes the exact numerical values, but not the trends that will be discussed below, as long as all the tone probability conditions that occurred in the experiment are allowed under the prior. These assumptions determine the joint probability *P*_*N*_*(past*,*future)* characterizing the statistical structure of the stimulus sequence, where *past* denotes the sequence of the last *N* stimuli and *future* denotes the next stimulus in the sequence (see Methods). The memory duration, *N*, is a parameter of the model and will be discussed in more details below.

As we discussed above, the most detailed (and complex) representation in this case would consist of a perfect encoding of the past sequence by identifying each of the 2^*N*^ possible configurations of the stimulation sequence with a unique state *m* (**Fig 2A**; case (i) is this representation for N = 4). This is, however, an unnecessarily detailed representation since the number of occurrences of the B tone among the last *N* is a sufficient statistic. The IB method identifies the relevant information for predicting the next stimulus: in our case, it assigns a single and unique state to all sequences that share the same number of occurrences of A tones and B tones **(Fig 2A;** case (ii)**).** This reduced representation filters out non-relevant details of the past (i.e. the exact order of the tones): it maintains the maximal predictive information possible (for a given *N*), but at a lower complexity (2.3 bits instead of 3.6 bits for *N* = 4). **Fig 2B** (gray line) displays the maximal predictive power at each complexity of the reduced representation for *N* = 4, and cases (i) and (ii) can be directly compared to each other.

Using the IB method enabled us to reduce the complexity of the representation even further. While representations that are simpler than the minimal sufficient statistic (case ii) do reduce the quality of the prediction of the next stimulus, the loss in predictive power may be relatively small. For example, for a memory of *N* = 4 tones, constraining the reduced representation complexity to 1 bit results in a ‘noisy’ representation with 3 unique states (**Fig 2A and 2B**, case (iii)). In this case, given the reduced representation alone, it is impossible to recover the exact number of B tones that occurred in the past (their exact order was already lost in the reduction to the minimal sufficient statistic). Nevertheless, this solution is optimal in the sense that its predictive power is maximal among all possible reduced representations with a complexity of 1 bit (for *N* = 4). For longer past durations and large complexities, the low sensitivity of predictive power to complexity is even more prominent. For a memory of *N* = 10 tones, the complexity reduction when using the sufficient statistic is larger than for *N* = 4, and further reducing the complexity of the representation by another 57% (from 3.46 to 1.49 bits) results in a loss of merely 12% in its predictive power (from 0.223 to 0.196 bits; **Fig 2B**, black).

**Fig 4:**
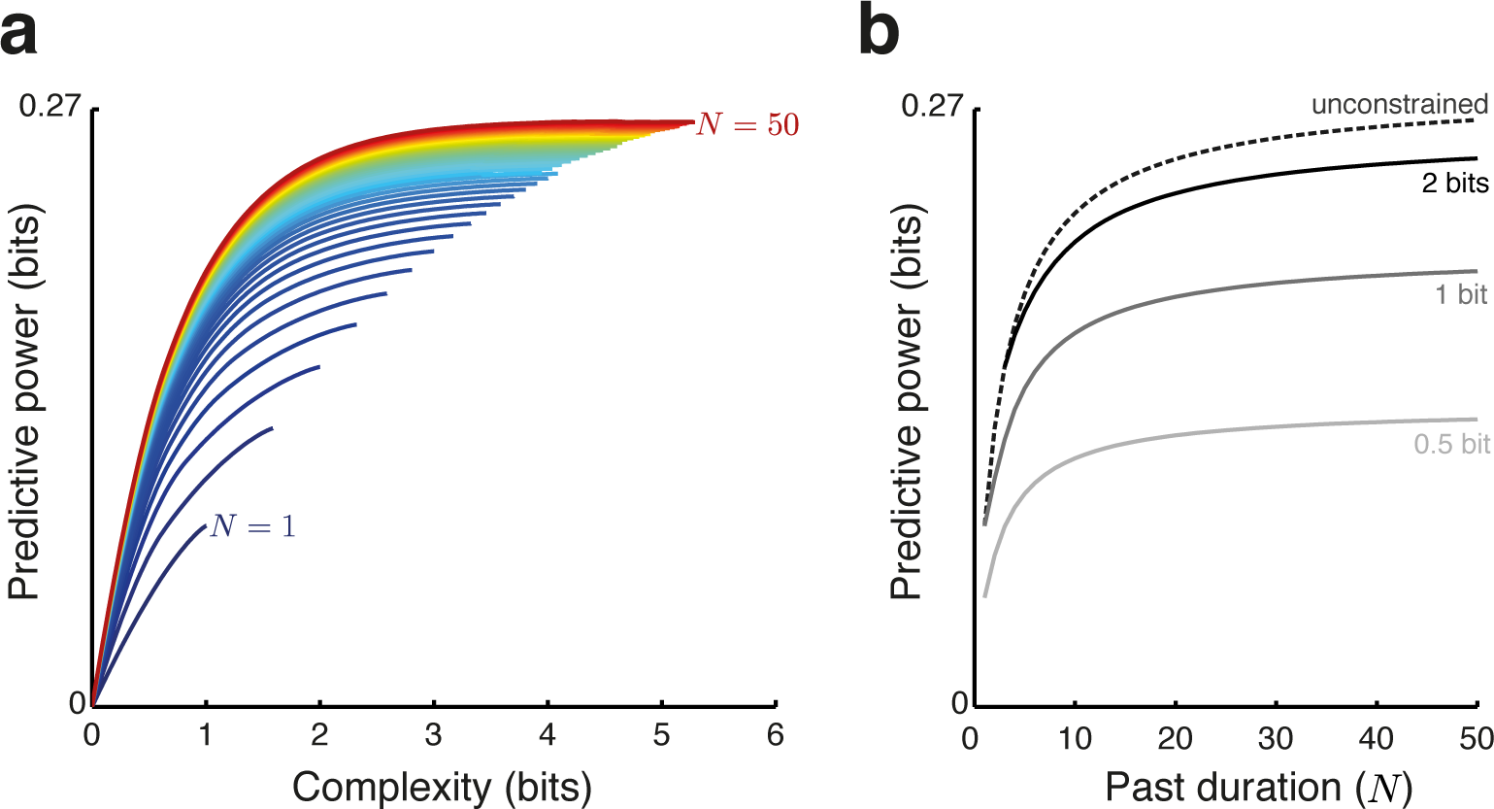
Tradeoff between complexity and predictive power in the oddball paradigm. (**a**) Tradeoff curves calculated for different durations of the past sequences, from *N* = 1 (blue) to *N* = 50 (red). The curves corresponding to *N =* 4 and *N =* 10 are the same as those shown in Fig 2. Each curve spans complexity values from 0 to that of the minimal sufficient statistic. While more complex representations exist, they cannot have higher predictive power. Each point along the curves represents one optimal solution (achieving maximal predictive power for its complexity constraint for the corresponding duration of the past). These curves separate achievable versus non-achievable combinations of complexity and predictive power (below versus above each curve, respectively). (**b**) The maximal predictive power that can be achieved as a function of the past duration *N*, for different constraints imposed on the complexity: 0.5 bit (light gray), 1 bits (dark gray), 2 bits (black). The dashed line corresponds to the predictive power of the minimal sufficient statistic, and is therefore the maximal predictive power at each past duration. Note the diminishing returns for increases in memory duration *N* (i.e. predictive power does not increase much beyond *N* = 10), as well as for increases in complexity (i.e. predictive power does not increase much beyond a complexity of 2 bits).

We examined the tradeoff between the complexity and the predictive power of the reduced representation for memory durations from *N* = 1 to *N* = 50 (**Fig 4A**) by applying the IB method to the probability distributions of the oddball paradigm *P*_*N*_*(past*,*future)*. Note that these probability distributions depend on N, the duration of the past. We used the IB algorithm to find, for each N, 200 reduced representations whose complexity spanned the full allowed range (see below), and whose predictive power was maximal for each value of the complexity. Each curve in Fig 4A is produced by linearly interpolating between these 200 points. The 200 points, however, sample the relevant ranges so densely that the linear interpolation is imperceptible. Thus, each point along the curves shown in Fig 4A corresponds to a unique optimal reduced representation: as described above, such reduced representation is given by the two conditional distributions *P(m|past)* and *P(future|m)*. The curves in Fig 4A plot the predictive power as a function of the complexity for these 200 reduced representations (the N = 4 and N = 10 cases are the same lines plotted in Fig 2B). These convex curves describe quantitatively the tradeoff between complexity and predictive power. Their convexity is a general property of the tradeoff postulated by the IB principle (see [13]).

For each *N*, complexity (abscissa of Fig 4A) spans the range between 0 and the complexity of the sufficient statistic. The predictive power (ordinate in Fig 4A) spans the range between 0 and the mutual information between past and future, which is equal to the mutual information between the sufficient statistic and the future. Thus, the rightmost point of each line shows the complexity of the sufficient statistic (abscissa) and the predictive power of the sufficient statistic (ordinate). More complex representations do exist (e.g. the full past), but do not result in an increased predictive power. As complexity is lowered below that of the sufficient statistic (moving leftward along a curve), predictive power is lost, but at least initially the loss of predictive power is rather minor.

The curves separate achievable and non-achievable combinations of complexity and predictive power. Any point on or below the curve is achievable, in the sense that there is (at least one) pair of conditional distributions *P(m|past)* and *P(future|m)* with the corresponding complexity and predictive power. Any point above the curve is non-achievable in this sense–there is no way to process the information from the past, keeping that level of complexity, and still get better predictions than those specified by the curve.

The dependence of optimal predictive power on *N*, the duration of the past, is illustrated in **Fig 4B** for different constraints imposed on the complexity (0.5, 1, 2 bits and with no constraints). Two effects are readily apparent. First, for each level of complexity, increasing past duration much above *N* = 10 does not result in a major increase in predictive power. Second, as hinted above, although the complexity of the sufficient statistic for long memory duration may be as high as 4–5 bits (**Fig 4A**), there is a strong effect of ‘diminishing returns’–complexity above 2 bits does not add substantial amount of predictive power to the reduced representation, even for a past duration of *N* = 50 stimuli (**Fig 4B)**. One way of understanding these effects is by noting that the predictive power reflects the precision of the probability estimates of the B tone. While this precision increases with both *N* and complexity, beyond a certain point increasing the precision at which the probability of the B tone is known does not improve much the predictions anymore.

### Neuronal Representation of Prediction Error

**Fig 5:**
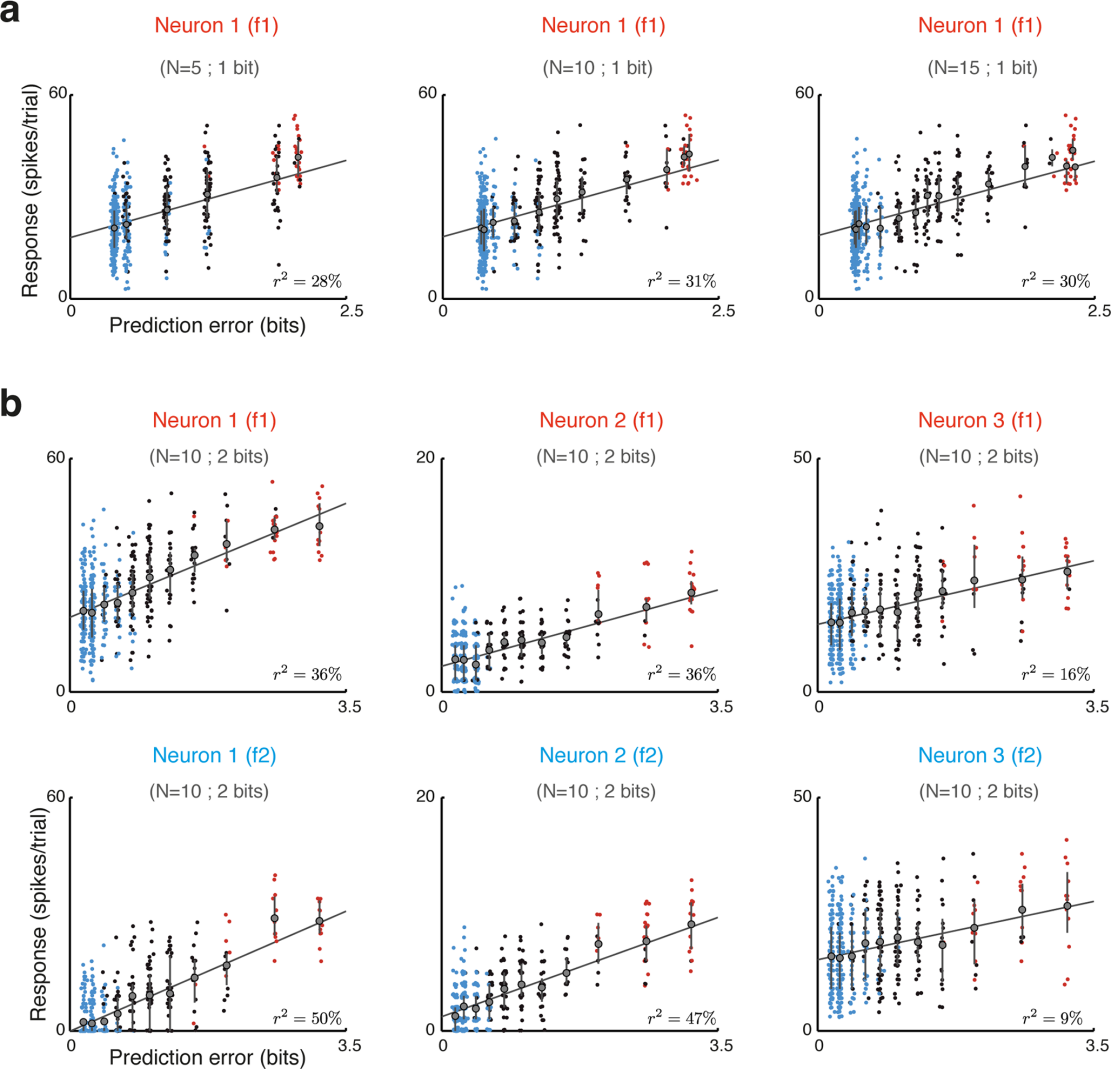
Neuronal representation of prediction error in the oddball paradigm. (**a**) Spike counts of single trial responses of one neuron to one of the two frequencies with which it was tested, plotted as a function of the expected prediction error for the same trial. The prediction errors were computed using optimal reduced representations at three past durations (*N* = 5, *N* = 10 and *N* = 15) with a complexity of 1 bit. A small amount of jitter was added to the *x* coordinate for visualization purposes only. Colors correspond to the different experimental blocks (*p* = 10% in red; p = 50% in black; p = 90% in blue). Error bars indicate the mean and the 25^th^ and 75^th^ percentiles for each value of the prediction error. (**b**) Responses of three different neurons (plotted separately for the two tones, top and bottom panels) as a function of the expected prediction error for a past duration of *N* = 10 stimuli at a complexity of 2 bits. Responses of the top leftmost panel belong to the same neuron shown in panel **a**.

To test the hypothesis that neurons in the auditory cortex represent the prediction error derived from a reduced representation of the recent past, we correlated the prediction errors, derived from the reduced representations we computed, with the neuronal responses to oddball sequences (**Fig 5**) [6, 20]. We calculated separately the prediction errors for each one of the reduced representations we computed above (defined by memory duration N = 1 to N = 50 and 200 complexity values ranging from 0 to the maximum possible for each N, for a total of 200*50 = 10,000 reduced representations).

For each reduced representation, for each neuron in the dataset, and for each of the two frequencies with which the neurons were tested, we used the actual sequence of tone presentations used in the experiment to calculate a corresponding sequence of prediction errors. We followed the prescription described above. For each tone presentation along the sequence, we used the previous *N* tone presentations as the past. Each past leads to a state *m* of the reduced representation, with an associated estimate *P(future|m)* for the probability of the upcoming stimulus. The prediction error associated with that stimulus was −log_2_ *P*(*future*|*m*). As explained above, the prediction error itself is a random variable. The transformation from past to reduced representation, *P(m|past)*, provided a set of probabilities to be in each of the states of the reduced representation, and through them to each of the possible prediction errors (see Methods). Therefore, instead of a single prediction error for each stimulus, we calculated a set of prediction errors, one for each possible state of the reduced representation *m*, together with their probabilities. To compare the prediction errors with the neuronal responses, we used linear regression of the neuronal responses against the prediction errors, weighted by the corresponding probabilities (see Methods for details). For illustration purposes, in **Fig 5** we plot neuronal responses (spike counts evoked by individual tone presentations, ordinate) against the average prediction error (−log_2_ *P*(*future*|*m*) averaged over all possible values for the state *m* of the reduced presentation, each with its probability *P*(*m*|*past*)) for that same stimulus presentation.

**Fig 6:**
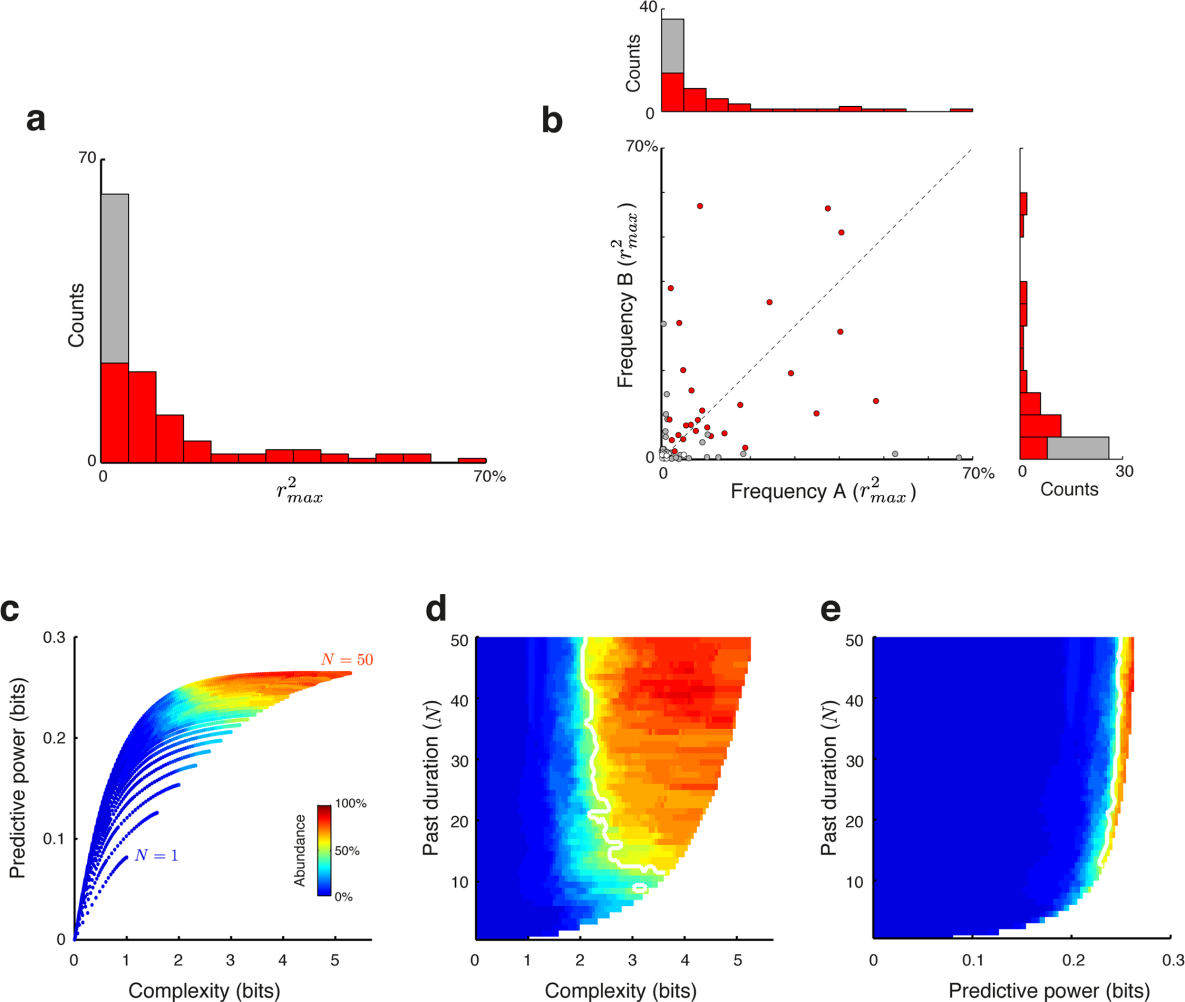
Population analysis. (**a**) Histogram of the best goodness-of-fit scores $r_{max}^{2}$ over the entire population (*n* = 117 combinations of 68 neurons × 2 test frequencies that evoked significant responses). Significant scores (permutation test, *p*<0.05; see Methods) are indicated in red (*n =* 78/117, 67%). (**b**) Scatter plot of the largest fractions of explained variance achieved for the two frequencies tested with each neuron in the main analysis (*n* = 68). Neurons with significant $r_{max}^{2}$ in both tested frequencies are indicated in red while neurons with significant $r_{max}^{2}$ in only one frequency are indicated in gray. (**c**) Two-dimensional, color-coded population analysis histograms of the complexity and predictive power underlying the ‘good representations’ (i.e., reduced representations that achieved at least 90% of $r_{max}^{2}$). Analysis was performed over combinations of neuron and frequency with $r_{max}^{2}\geq 0.1$ (*n* = 34/117). Color scale (from blue to red) represents the fraction of neurons for which that combination was ‘good’. An abundance of 100% (red color) means that this combination of parameters was in the ‘good’ set of parameters for each and every neuron in the analyzed population. (**d**) Same analysis as in panel **c**, results are shown as a function of past duration and complexity. The 50% contour in the plot is marked by the white line. **(e)** Same analysis as in panel **c**, results are shown as a function of past duration and predictive power. Panels **d** and **e** use the same color-code as panel **c**.

In the experiments, neurons were tested using blocks composed of two tones whose frequency separation was about half octave (f_high_/f_low_ = 1.44; this is the Δf = 0.37 case of [6, 20]). The probabilities of the two tones in each block were fixed, but their order was random. For the main analysis (Figs 5 and 6), we used a conservative counting window (0–330 ms after stimulus onset; tone duration was 230 ms and onset-to-onset interval was 730 ms), and we used the responses of all 68 neurons tested with 3 blocks in which the tone probabilities were 90%/10%, 50%/50%, and 10%/90% (for the low frequency and high frequency tones respectively). The use of responses from these 3 blocks ensured sampling of the full range of relevant values of prediction errors. The analysis was performed separately for each frequency, resulting in 136 combinations of neuron and frequency, with 117/136 combinations showing responses to the corresponding tone that were significantly larger than the spontaneous rate.

Prediction error was significantly correlated with neuronal responses in many cases. **Fig 5A** compares responses of one neuron (spike counts evoked by single presentations of one of the two frequencies with which it was tested) with the expected prediction errors calculated for each tone presentation using three different optimal reduced representations. These representations had a complexity of 1 bit and past durations of *N* = 5, *N* = 10 and *N* = 15 stimuli. Each block had 400 stimuli, of which 90% (360), 50% (200) or 10% (40) consisted of the relevant frequency. To avoid using stimuli whose past sequence was not well defined, the first 50 stimuli of each block were removed from the analysis. Due to the randomized nature of the sequences, for each combination of neuron and test frequency we ended with 311–322 stimuli in the p = 90% condition (blue points), 168–182 stimuli in the p = 50% condition (black points), and 28–39 stimuli in the p = 10% condition. As the tones presented in the 10% condition were, by definition, rare, they were associated with large prediction errors, and therefore the red points are mostly concentrated at the right of the scatter plots. Similarly, the tones presented in the 90% condition were, by definition, common, and were associated with small prediction errors. Therefore the blue points are mostly concentrated at the left of the scatter plots. On the other hand, for the 50% condition, prediction errors varied quite substantially, leading to a larger dispersion along the abscissa between (and partially overlapping) the two extremes.

While the correlation with the prediction errors corresponding to *N* = 10 was slightly larger than for shorter or longer memory durations, this example mainly illustrates the finding that the correlation between the prediction error and neuronal responses was sometimes only weakly dependent on past duration, a finding we will return to later. To illustrate the range of goodness of fit that could be achieved, Fig 5B compares single-trial responses of three neurons (columns; the rows show the two frequencies used for testing each neuron) versus their expected prediction errors, calculated using reduced representations with complexity of 2 bits and past duration of *N* = 10 stimuli. Neurons 1 and 2 had strong dependence of their responses on prediction error, while Neuron 3 was typical for the data.

To quantify these observations for the entire data set, we performed weighted linear regression analysis, separately for each combination of a neuron and frequency that evoked significant responses (*n* = 117 combinations), using the entire set of pre-calculated optimal solutions. The distribution of the resulting best goodness-of-fit scores ($r_{max}^{2}$) is displayed in **Fig 6A**.

We tested the significance of the fit separately for each combination of neuron and frequency. The goodness of fit scores were calculated as the maximum *r*^2^ over all possible reduced representations, so that standard significance tests could not be applied due to the inherent multiple comparisons involved. We therefore tested the significance of a fit using a permutation test. We repeated the same analysis for the measured spike counts but replacing the stimuli by 20 randomly permuted sequences with the same tone probabilities, thus breaking the short-term relationships between responses and associated prediction errors. The effect of prediction error on the neuronal responses was considered to be significant (*p*<0.05) if $r_{max}^{2}$ for the actual stimulation sequence was larger than $r_{max}^{2}$ calculated using 20 different random permutations. The prediction error significantly influenced the neuronal responses in about 2/3 of cases (78/117 combinations, 67%; see **Fig 6A**, red bars), supporting our hypothesis that auditory cortical neurons represent this formal notion of prediction error in their responses.

Most neurons contributed to **Fig 6A** twice, so the figure could overstate the degree to which prediction error and neuronal responses are related to each other. **Fig 6B** is a scatter plot of the goodness of fit values for the two frequencies, with the corresponding histograms for the two frequencies separately. There was a mild correlation between the goodness of fit values for the two frequencies (r = 0.34, n = 68, *p*<0.05, using the normal approximation to the Fisher z-transformed correlation), suggesting that the representation of prediction error in the neuronal responses was at least to some degree a property of the neuron itself. Considering each frequency by itself, 41/62 neurons that responded significantly to the low frequency and 37/55 of those that responded significantly to the high frequency showed a significant dependence of neuronal responses on prediction error. More conservatively, 52 neurons had significant responses to both frequencies, and 27 of these neurons showed significant dependence of the responses to both frequencies on prediction error. Thus, depending on the criterion, between 1/2 and 2/3 of the neurons showed significant dependence (*p*<0.05) of the neuronal responses on prediction error.

In order to characterize the past duration and the complexity of the representations that best fitted the neuronal responses, we analyzed the parameters of these representations that best explained the data. For this analysis we used only combinations of neuron and test frequency with explanatory power of $r_{max}^{2}\geq 0.1$ (adjusted correlation coefficient > 0.33, *n* = 34/117, see Methods). We found that the dependence of the goodness of fit scores on the parameters (memory duration and complexity) could be rather weak once either past duration or complexity were large enough, so that many different models achieved approximately the same goodness of fit. Therefore, we defined, for each combination of neuron and test frequency, a set of *good representations* consisting of those that achieved goodness-of-fit scores higher than an (admittedly somewhat arbitrary) threshold: $r^{2}\geq 0.9∙r_{max}^{2}$. The parameters of these ‘good representations’ (past duration, complexity and predictive power) are summarized in two-dimensional, color-coded histograms (**Figs 6C–6E**). For each reduced representation, the histograms show the fraction of cases for which that specific representation belonged to the set of good representations. The reduced representations are index in three different ways (by complexity and predictive power, Fig 6C; complexity and duration, Fig 6D; predictive power and duration, Fig 6E). Thus, Fig 6D shows that reduced representations with duration *N* < 10 stimuli were outside the set of good representations for most cases. Thus, for a reduced representation to be good for a large fraction of cases, it had to have a relatively long past duration, *N* ≥ 10 stimuli (corresponding to 7.3 seconds or longer). Similarly, Fig 6D shows that for a reduced representation to be good for a large fraction of cases, it could have a complexity as low as about 2–3 bits (white line in Fig 6D)–reduced representation with lower complexities tended to be outside the set of good representations for most cases. Moreover, although the resulting reduced representations were relatively coarse (low complexity) their predictive power almost matched the predictive power attainable with models that have maximal complexity (**Fig 6C** and **6E**; compare with **Fig 4A** and **4B**). Thus, the good reduced representation with shortest memory tended to have a long memory (N>10), coarse (complexity of 2–3 bits relative to maximal complexity of ~5 bits), but keep a high degree of predictive power.

### Controls and Extensions

#### Prediction errors and single-block trial-by-trial variability

The previous analysis demonstrated that prediction error had a substantial effect on the responses of many neurons. However, the responses were collected from three different experimental blocks that had different overall probability for the tone, and therefore different levels of typical prediction error. Since we have already shown that the average responses depended on overall probability (**Fig 2**), the correlation between responses and prediction errors could be solely due to the difference between the average responses to the same tone in the three blocks in which it was tested, and not to trial-by-trial fluctuations of the prediction error within one block.

To verify that trial-by-trial response variability reflected variation in the prediction error, we repeated the above analysis confined to individual blocks. Neurons were tested in five probability conditions: 10%, 30%, 50%, 70% and 90%. All neurons in the dataset (n = 99) were tested in the 10% and in the 90% conditions. A subset (n = 68) was also tested in the 50%/50% condition (these are the neurons used in the main analysis, **Figs 5** and **6**), and a subset of those (n = 29) was tested in two additional probability conditions, 70%/30% and 30%/70%.

For interpreting the results of these analyses, it should be kept in mind that the range of prediction errors considered in the main analysis was larger than the range of prediction errors within each block. For example, in the 90%/10% block, the probable tone was not very surprising–it occurred on average 90% of the time in each segment of *N* stimuli. Because of the random nature of the sequence, there were nevertheless small fluctuations in the overall number of the two tones within each block of *N* stimuli, leading to small fluctuations in the prediction error. On the other hand, the variability of the responses, conditioned on the prediction error computed from the reduced representations (essentially the width of the scatter around the regression lines in the examples of Fig 5), remained roughly the same within block as it was across blocks. Thus, the fraction of explained variance of the responses by the prediction error (and therefore the significance of the dependence of the responses on prediction error) was expected to be smaller in the within-block analysis than in the main analysis. Formally, assume *Y* = *aX* + *b* + *n*, where *Y* are the responses, *X* the prediction errors, *a* and *b* are the regression coefficients (assumed perfectly known here for simplicity), and *n* the noise around the regression line, usually assumed to be independent of *X*. The explained variance is

$$1-\frac{var(n)}{var(aX+b+n)}=1-\frac{var(n)}{a^{2}var(X)+var(n)}$$

so that the explained variance is a monotonic function of var(*X*). In the within-block analysis, var(*X*) is smaller than in the main analysis, and therefore the explained variance is expected to decrease.

**Fig 7:**
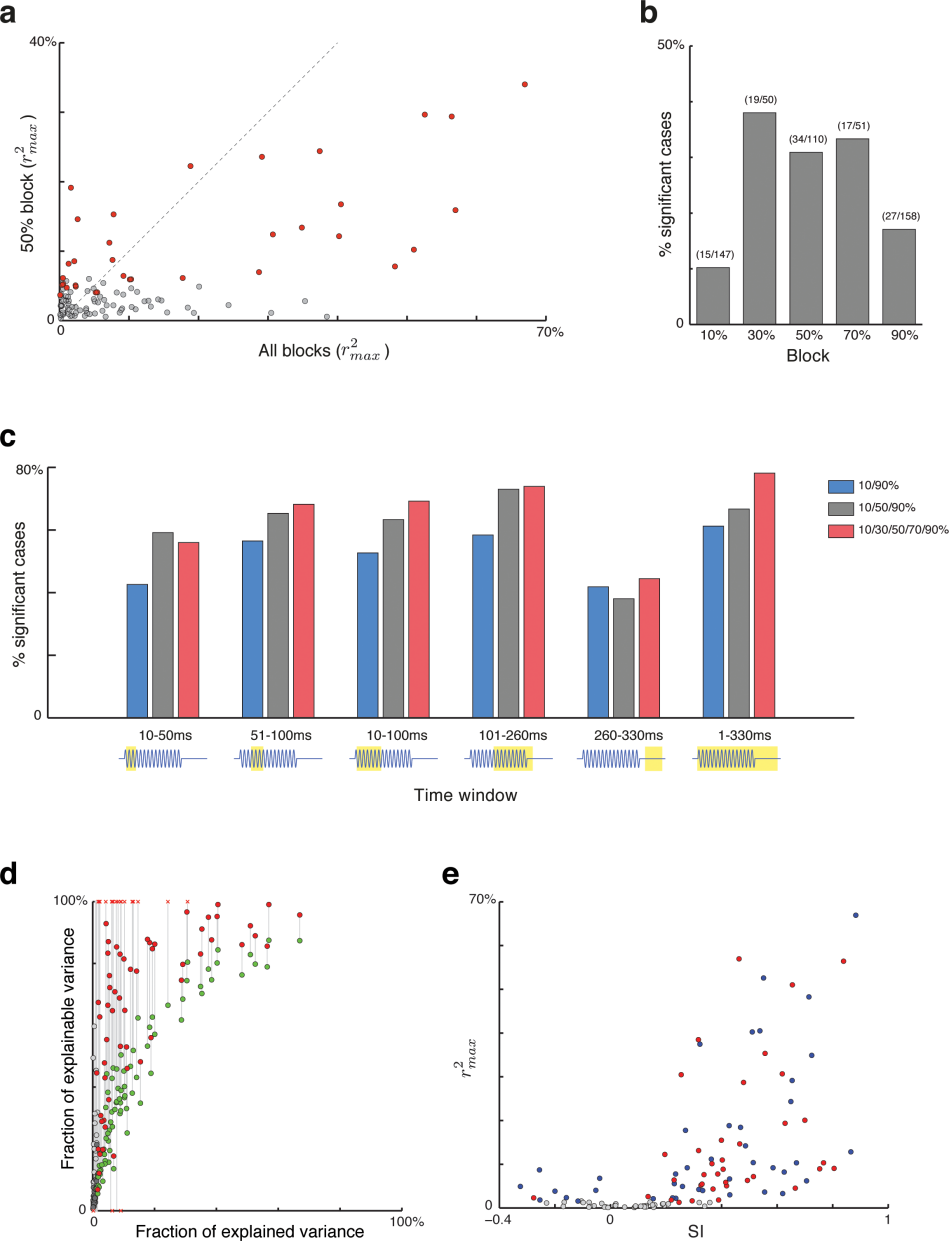
Controls and extensions of the main analysis. (a) Comparison of the best goodness-of-fit scores achieved for each combination of neuron and test frequency that had significant response in the ‘equiprobable’ block (p = 50%) and the goodness-of-fit score of the same neuron and frequency in the main analysis (using all responses with p = 10%, 50% and 90%). Significant scores in the equiprobable block are indicated in red (permutation test, *p*<0.05). (b) Fraction of cases with significant effects of prediction error on the neuronal responses for all the single-block analyses. (c) Fraction of cases with significant effects of prediction error on the neuronal responses in different time windows (marked below the histogram) and for different selection of blocks. (d) Comparison of the fraction of explained variance ($r_{max}^{2}$) and the fraction of explainable variance. The red points corresponds to correction by a noise estimates using unbiased variances, while the green points correspond to the conservative correction by the (smaller) noise estimates using the biased variances. (e) Comparison of SSA indices (SI) and explained variance ($r_{max}^{2}$). Cases with significant correlation (permutation test, *p*<0.05) are indicated by blue (low tones) or red (high tones).

In the equiprobable block the prediction error spanned the largest range of values (var(*X*) was largest in the notation above). The $r_{max}^{2}$ scores of each combination of neuron and frequency in the single-block analysis are plotted in **Fig 7A** against the corresponding value for the main analysis (that included in addition the 10%/90% and 90%/10% blocks). Only combinations of neuron and frequency with significant neuronal responses within the equiprobable block are shown in this analysis (n = 110/136 combinations). Significant correlations (*p*<0.05, see Methods) of prediction errors and neuronal responses were found in 34/110 combinations of neurons and frequencies with significant responses (31%; see **Fig 7A**, red dots). As expected, the fraction of explained variance was generally smaller for the responses restricted to the equiprobable block than for the corresponding data in the main analysis.

We performed the single-block analysis for all probability conditions. **Fig 7B** shows the fraction of combinations of neuron and frequency that showed a significant effect of prediction error on the neuronal responses (out of the combinations with significant responses in each condition). The substantial number of cases with significant dependence of neuronal responses on prediction error confirms that prediction error could account for trial-by-trial fluctuations within blocks as well as across blocks. The larger number of significant cases found in the mid-range probability conditions may be due to the larger range of values spanned by the prediction error in these blocks, increasing the power of the statistical test.

#### Prediction error and response time course

Ulanovsky et al. [6, 20] showed that the dependence of the responses on context developed over time–it was maximal about 100 ms after stimulus onset, and somewhat weaker around stimulus onset and offset. The analysis window used here (0–330 ms after stimulus onset) contained all response components, including the onset, sustained and offset components. We therefore repeated the same analyses using different time windows for counting spikes. In all cases, combinations of neurons and tones that had significant responses in that time window were selected for statistical evaluation. **Fig 7C** displays the fraction of combinations of neuron and frequency that showed significant effects of prediction error in the different time windows, for various combinations of probability conditions. Gray bars correspond to the use of the 90%/10%, 50%/50% and 10%/90% blocks as in the main analysis. The fraction of cases that showed significant effects of the prediction error on neuronal responses when using these blocks was mostly between 0.6 and 0.7, except for the offset time window (260–330 ms after stimulus onset, stimulus duration was 230 ms). Still, even for this late time window, over half the cases (71/117) had significant responses, and in about 40% of these cases, there were significant effects of prediction error on the neuronal responses. The average fraction of explained variance in the late time window was, however, significantly smaller than while the stimulus was on, even after selecting only cases with significant effects of prediction error (linear mixed effects model, time window with random unit effect; effect of time window: F(3,215) = 5.66, p = 0.00095; post hoc comparisons with *p*<0.05). We repeated this analysis using the 10% and 90% conditions only (**Fig 7C**, blue bars) and also using the all probability conditions– 10%, 30%, 50%, 70% and 90% (**Fig 7C**, red bars), with a very similar pattern of results.

#### Effects of noise variance

We address here the observation that even when prediction error significantly modulated the responses, the fraction of explained variance accounted for by the prediction errors was often rather small. We return here to the conditions of the main analysis: using only three probability conditions (10%, 50% and 90%) with the full counting window (0–330 ms after stimulus onset).

The fraction of explained variance is the ratio between the predictor variance (that part of the overall variability of the spike counts that is accounted for by the linear fit to the prediction errors) and the overall variance of the spike counts. We used an approach introduced by Sahani and Linden [25] and recently used by others [26, 27]. In this approach, the overall variance is partitioned into two parts. One part is the noise variance–the variability of the spike counts that cannot be accounted in any way by the experimental manipulation. The other part is the explainable variance (‘signal power’ in [25]), which consists of those aspects of the spike counts that are reproducible given the stimulation sequence. The fraction of explained variance may be small because the noise variance is large rather than because the model is inadequate. Thus, for assessing the quality of a model, it makes sense to compare the explained variance with the explainable variance only, ignoring the noise, which is experimentally uncontrolled. Such calculation produces a ‘fraction of explainable variance’, in contrast with the fraction of explained variance, $r_{max}^{2}$, used throughout the rest of the paper.

Since we study the effect of stimulus context on neuronal responses, the noise variance is the variance of the evoked spike counts conditioned on the exact sequence of previous stimuli. Such noise variance is often computed from the responses evoked by the same long stimulus sequence presented many times (e.g. Sahani and Linden 2003). Unfortunately, such data were unavailable here. We therefore used an approximation.

We really have to estimate the variance of the spike counts conditioned on the previous long stimulus sequence (e.g. 50 stimuli back, as long as the longest memory duration we used for calculating the prediction errors). There are however 2^50^ ≈ 10^15^ such sequences, and each neuron was tested with only about 10^3^ individual tone presentations (distributed over separate experimental blocks), each of which sampled one of the possible sequences of preceding 50 stimuli. This sparse sampling made it impossible to directly estimate noise variance.

Instead of conditioning the variance on the 50 preceding stimuli, we conditioned the variance on the preceding 7 stimuli, resulting in at most 128 different past sequences, but in addition we conditioned on the block in which the sequence occurred. By conditioning on the block, we took at least partially into account the differences in the more remote past between the same short (7 stimulus long) contextual sequences as they occurred in different probability conditions. We ignored the initial 50 stimuli from each block, since they were omitted from the main analysis. Our estimate of the noise was a weighted average of the variances of each such set of responses (the ‘conditional variance’, with identical preceding sequence of 7 stimuli, computed for each probability condition separately). The weights were the number of responses used to calculate each conditional variance, so that conditional variances that were estimated better (from more responses) had proportionately greater representation in the estimate of the noise. Finally, we calculated the fraction of explainable variance by dividing the predictor variance by the explainable variance, calculated by subtracting the estimate of the noise variance from the overall variance of the spike counts.

We used two estimates of the conditional variance. The first used the unbiased variance estimated for each set of responses (sum of squared deviations from the mean of the set, divided by n-1). To be conservative, we also used the biased estimate of the variance (dividing the sum of squared deviations from the mean by n rather than by n-1). This reduced the estimates of the conditional variances, and ensured that the estimated noise variance was always smaller than the overall variance (which is not always the case when using the unbiased estimate of the variance).

Fig 7D shows a scatter plot of the fraction of explained variance ($r_{max}^{2}$ as used previously, abscissa) and the fraction of explainable variance (ordinate). The red points corresponds to the use of the unbiased conditional variances, while the green points correspond to the use of the (smaller) noise estimates using the biased conditional variances. Some of the corrected values using the unbiased variance estimates were negative (markers at the bottom of the plot) or greater than 1 (markers at the top of the plots). Negative estimates correspond to cases in which the unbiased estimates of the noise were larger than the actual variance of the counts. Estimates of fraction of explainable variance that were larger than 1 correspond to cases in which the noise estimate was smaller than the overall noise variance (as it should be) but the remainder was smaller than the predictor variance, suggesting that the noise estimates in these cases also were too large. When using unbiased noise estimates, such cases of over-correction are expected to occur. On the other hand, the conservative estimates of fraction of explainable variance were highly correlated with the uncorrected estimates and all of them were positive and smaller than 1, suggesting that the conservative noise estimates consistently underestimated the true noise variance.

Even when under-corrected (green points), the fraction of explainable variance was consistently larger than the uncorrected fraction of explained variance, showing that noise variance had a substantial effect, weakening the strength of the linear relationships between prediction error and neuronal responses. Table 1 summarizes the results.

**Table 1:** Comparing fractions of explained and explainable variance.

Fraction of:	Explained variance	Explainable variance, conservative	Explainable variance, unbiased
>0.1, significant	34/78, 44%	73/78, 94%	74/78, 95%
>0.1, non-significant	0/39, 0%	3/39, 7.7%	17/39, 44%
>0.64, significant	1/78, 1.3%	16/78, 21%	54/78, 69%
>0.64, non-significant	0/39, 0%	0/39, 0%	4/39, 10%

Significant and non-significant refer to the effect of prediction error; there were 78/117 cases with significant effects, 39/117 with non-significant effects.

For both correction schemes, almost all cases with significant effects of prediction error achieved a fraction of explainable variance greater than 0.1 (correlation coefficient of 0.33). The unbiased correction had also a large increase in the number of cases with non-significant effects of prediction error that achieved a fraction of explainable variance greater than 0.1; we don’t believe that these cases are necessarily significant–rather, this increase probably reflects the weaker statistical stability of this scheme.

More importantly, both schemes increased substantially the number of cases with large fraction explainable variance. We use explainable variance > 0.64 (correlation coefficient of 0.8) as an arbitrary cutoff, but the pattern of results does not depend on this specific cutoff. Only one case achieved larger explained variance (with no correction for noise variance). However, 16 and 54 cases (out of 78) achieved a fraction of explainable variance larger than 0.64 using the two correction schemes. Only 0 and 4 (out of 39) non-significant cases achieved such high fraction of explainable variance. These calculations suggest that in more than half the cases, the fraction of explainable variance by a linear fit to the prediction error was substantial (at least greater than 0.5).

Nevertheless, even in the best cases, the fraction of explainable variance didn’t seem to fully saturate by the linear fits to the prediction errors. The failure to saturate the explainable variance could reflect the existence of non-linear relationships between prediction errors and neuronal response. For example, a sigmoidal or a threshold-linear relationship could fit better the data. Because of the additional complexity of these models, we did not explore them further here.

#### Prediction errors and SSA indices

In the large majority of studies of stimulus-specific adaptation in the auditory system, the strength of the adaptation is quantified using the contrast between average standard and deviant responses (e.g. Ulanovsky et al. 2003, Reches and Gutfreund 2008, Malmierca et al. 2009), called the SSA index or SI. The goodness of fit used here, measured by $r_{max}^{2}$, is a different measure of the difference between the responses to standards (with small prediction errors) and deviants (with large prediction errors). However, these two measures take into account somewhat different aspects of the responses–while the SI depends only on the average responses to standards and deviants, $r_{max}^{2}$ is also sensitive to the variability around these means. In consequence, a large SI may come with a small $r_{max}^{2}$ because response variability is large, and a small SI may come with a large $r_{max}^{2}$ in case the variability in the responses is much smaller than the difference between the standard and deviant responses.

Fig 7E is a scatter plot of the SI (abscissa) and $r_{max}^{2}$ (ordinate), illustrating these general comments. Thus, for example, cases with SI>0.6 had $r_{max}^{2}$ spanning the range from very small (below 0.1) to the largest achieved in this sample (close to 0.7). Similarly, cases with reasonable large $r_{max}^{2}$, for example larger than 0.4, had SIs spanning the range from about 0.3 to 1. In conclusion, neither the SI nor the $r_{max}^{2}$ fully characterize the context sensitivity of a neuron–both supply useful, to some extent independent, information about it.

## Discussion

We present here an information theoretic formulation for the problem of sensory perception and prediction in the brain. We suggest that the representation of past stimuli reflects a tradeoff between the complexity of the reduced representation and its predictive power. The prediction errors, calculated from the statistics of the stimulation sequence using first principles, were significantly associated with the measured neuronal responses.

Importantly, these results suggest that the relevant time scale for the calculation of the prediction error is very long relative to the time scale usually considered in sensory coding–on the order of several seconds or longer. Our results therefore suggest that neurons in auditory cortex rely on surprisingly long time scales to calculate prediction errors, although the representations of the past, which underlie the computation of the prediction errors over these time scales, were found to be rather coarse.

Attneave [28] and Barlow [29] already suggested that neural information processing might follow principles of information theory. Information theory provides universal bounds on the minimal expected prediction error that can be achieved, independent of other assumptions on the implementation of a predictive process by the brain. In this sense the theory is normative: it specifies absolute bounds that cannot be improved in any way, and that are achieved by specific reduced representations of the past sequence. These bounds are governed by only two parameters, the complexity of the reduced representation (or, alternatively, its predictive power) and the duration of the past memory used for perception.

The notion of predictive coding is not new. The importance of the interplay between the representation complexity and the accuracy of predictions has been noticed before (e.g. [12]). In recent years, the tradeoff has been used extensively by Friston [30]. Friston used an approximation to the exact Bayesian inference problem, which is very difficult. Our approach circumvent this difficulty: crucially, it differs from previous attempts to formally use the interplay between complexity and prediction quality in that the prediction error in our formulation is derived from a reduced representation rather than directly from the explicit past stimulus sequence. Most importantly, one unique aspect of our approach is that we use a model-independent (information theoretic) bound.

In spite of the generality of this approach, we could apply it to real experimental situations: we show how to use it for studying rigorously the neuronal code in one simple case. It turns out that the neural responses reflect the prediction error derived from efficient reduced representations, allowing us to extract bounds on the duration of the memory that underlie the observed neuronal responses as well as on its complexity: memory duration is remarkably long (longer than 10 stimuli back) but rather coarse (with a complexity less than 2 bits).

The theory presented here can be considered as part of a more general theory of neural function. Since the reduced representation is assumed to be internal to the organism, its complexity may be related to the metabolic cost required to maintain and update it. As we illustrated in the case of the oddball sequences, less complex representations in our sense are also simpler to implement biologically–they require a smaller number of states, and incorporate noisy assignments of past sequences to states of the representation. On the other hand, constraints on expected prediction error may stand for general constraints on future value. Thus, our theory can be seen as an application of the general principle that organisms attempt to minimize metabolic or other costs subject to future value constraints. This principle unifies most known learning and control theoretic models, potentially linking information theoretic measures with general biological constraints directly [31].

Our approach provides a principled way to study long-term dependencies in neuronal responses, which are inaccessible to many models of auditory responses [32–34]. These models relate the neuronal responses with the preceding content of the stimulus within a short time window (typically 50–100 ms). Attempts to include context in such models end up with analyzing relatively short-term contextual effects, from a few ms [16] to a few tens of ms [35] and up to a few hundreds of ms [36, 37]. Only a few attempts to quantify longer-range dependencies have been published. For example, Ulanovsky et al. [20] demonstrated (in a previous analysis of the data presented here) that the larger responses to rare tones could not be accounted for by taking into account only a recent past (*N* ≤ 4 preceding stimuli, 3 seconds). They concluded that the larger responses to rare stimuli depended on longer segments of the stimulation sequence, although the effective memory duration and its content were left unspecified in these studies. Similarly, studies that fitted more mechanistic models of synaptic depression to such data [19, 23] concluded that such models cannot fully account for the data, but such conclusions were limited by the restricted range of models that have been considered.

Our main experimental observation in this paper is that the dependence of neuronal responses on prediction error calculated over long time scales can be made precise. This observation supports the notion that auditory cortex neurons carry a prediction error signal. Furthermore, we specify for the first time potential properties of the reduced representation underlying auditory cortical responses to oddball sequences at such long time constants. In particular, we demonstrate that the neuronal responses are compatible with reduced representations that have long memory duration but low complexity.

Importantly, the descriptive power of our theory is not restricted to statistically simple sequences such as the oddball paradigm presented here. The full power of the theory resides in more complex stimulation sequences that may lack sufficient statistics (e.g. [21]). Of particular interest are the statistical regularities related to the syntax and semantics of language and music, which span multiple temporal scales. The tools developed here allow for a formal examination of the sensitivity of neurons to the complex statistical regularities of real-world soundscapes and therefore present a broad framework for characterizing sensory perception both qualitatively and quantitatively.

## Methods

### Electrophysiology and Stimulus Presentation

The neuronal responses are those described in [6, 20], which also contain the detailed experimental methods. In brief, extracellular recordings were made in primary auditory cortex (A1) of halothane-anesthetized cats. Anesthesia was induced by Ketamine and Xylazine and maintained with halothane using standard protocols authorized by the committee for animal care and ethics of the Hebrew University—Hadassah Medical School. Single units were spike sorted on-line using template-based sorting, and in most cases they were also sorted off-line, to improve unit isolation. Stimuli were presented to the animal through sealed, calibrated earphones. For the oddball paradigm, two frequencies were selected close to the best frequency of the neuron, with a frequency ratio f_high_/f_low_ = 1.44. Ulanovsky et al. (2003; 2004) defined the frequency difference slightly differently, and this is their Δf = 0.37 condition. The two frequencies were presented in a number of blocks. Each block contained 400 pure tone stimuli of identical duration (230 ms), inter-stimulus interval (736 ms onset to onset) and tone level (approximately 40 dB above the neuron’s minimal threshold). The blocks differed by the number of times each frequency was presented. For example, in the 90%/10% block, 360 of the stimuli had frequency f_low_ and 40 had frequency f_high_, presented using a random permutation. We also used blocks with probabilities 70%/30%, 50%/50%, 30%/70%, and 10%/90%. The dataset was composed of 99 neurons tested in the 90%/10% and the 10%/90% cases. Of these, 68 were also tested in the 50%/50% (these are the neurons used in the main analysis); and of those 68 neurons, 29 neurons were additionally tested in the 70%/30% and 30%/70% conditions.

### The Information Bottleneck (IB) Method

For a detailed presentation of the IB principle & algorithm see Tishby et al. [13]. In short, given a joint distribution *P(x*,*y)*, the IB finds a *compressed* representation of *x* denoted by *m* that is most *informative* on the target variable *y*. The compression of the representation is quantified by the mutual information between *x* and *m*, given by *I(x;m)*, and the information that *m* carries on the target variable *y* is quantified by *I(m;y)*. Using the IB algorithm we effectively pass the information that *x* provides about *y* through a ‘bottleneck’ formed by the reduced representation *m*, defined by *P(m|x)*. In practice, the reduced representation is determined by minimization of the Lagrangian, L[P(m|x)]=I(x;m)−βI(m;y) with respect to *P(m|x)*. The positive Lagrange multiplier *β*, associated with the constraint on *I(m;y)*, controls smoothly the tradeoff between preserving relevant information and the compactness of the representation. The optimal assignment that minimizes the IB Lagrangian satisfies the following equations:

$$P(m|x)=\frac{P(m)}{Z(x,\beta )}exp(-\beta D_{KL}[P(y|x)||P(y|m)])$$

$$P(m)=\sum_{x}P(x)P(m|x)$$

$$P(y|m)=\sum_{x}P(y|x)P(x|m)$$

where *Z(x*,*β)* is a normalization (partition) function. We used here an iterative algorithm (over the set of self consistent equations) to find the optimal solution *P(m|x)* for a given *P(x*,*y)* and *β*. In our case, *x* and *y* are the past and future of the stimulus, respectively, and *m* is the reduced representation of the past.

### Reduced Representations in the Oddball Paradigm

We computed reduced representations for the oddball paradigm, for a given past duration of *N* stimuli. First, we calculated the joint distribution *P*_*N*_*(past*,*future)* corresponding to the oddball sequences. For any given value of the parameter *p* (the probability of the ‘high tone’ in the block) the events are independent by construction. Thus, for a uniform prior over *p*, the posterior probability can be calculated explicitly,

PN(past,future=high)=∫01(Nk)pk+1(1−p)N−kdp=k+1(N+1)(N+2)

where *k* = 0,1,..,*N* indicates the number ‘high tones’ in *past*. Then we applied the IB method over *P*_*N*_*(past*,*future)* to find reduced representations of the past that are predictive with respect to the future (i.e., the next stimulus). We repeated this procedure with 200 different values of *β* to span the entire range of complexity and predictive power levels, and with 50 different values of *N* spanning past durations from *N* = 1 to *N* = 50. We ended up with a set of 200 × 50 reduced representations, where each one was defined through the conditional probability distributions: *P(m|past)* and *P(future|m)*.

### Prediction Errors

We used the conditional probability distributions *P(m|past)* and *P(future|m)* to calculate prediction errors along the stimulation sequence, as follows. For each stimulus (*future*) we considered its previous *N* stimuli (*past*) to determine the probability of entering each state *m* of the reduced representation using *P(m|past)*. The probability with which the future stimulus is then expected *P(future|m)* was used to calculate the prediction error −log_2_ *P*(*future*|*m*) associated with that stimulus. Since this quantity depends on the unknown state *m*, we used the *expected* value of the prediction error (with respect to *m*) to generate Fig 5:

$$E(future|past)=-\sum_{m}{P(m|\mathrm{past})\log}_{2}P(future|m)$$

Note that the expected prediction error with respect to the stimulus distribution *P(past*,*future)* is negatively related to the predictive power, *I(m;future)*:

$$\begin{array}{l} {\left\langle E(future|past)\right\rangle }_{P(past,future)}\\ \;=-\sum_{past,m,future}P(past,future)P(m|past)\log_{2}P(future|m)\\ \;=-\sum_{past,m,future}P(past,m,future)\log_{2}P(future|m)\\ \;=-\sum_{m,future}P(m,future)\log_{2}P(future|m)=H(future|m)\\ \;=H(future)-I(m;future) \end{array}$$

where *H(Future)* is the entropy of the future stimulus and does not depend on the reduced representation. It follows that maximizing the predictive power is equivalent to minimizing the expected prediction error.

### Fitting Neural Data

For the main analysis, spike counts were measured in a window of 330 ms, starting at stimulus onset and ending 100 ms after stimulus offset. For the analysis displayed in **Fig 7**, shorter windows were used as well. We collected the neuronal responses (represented as spike counts) for each neuron and for each test frequency (‘low’ or ‘high’) across the oddball stimuli blocks.

For each combination of neuron, test frequency, and reduced representation (200 × 50 pre-calculated reduced representations) we calculated the prediction error at each stimulus along the actual stimulation sequence used in the experiments. Since the prediction error is calculated with respect to a past window of up to *N* = 50 stimuli, the first 50 stimuli in each block were omitted from the analysis, resulting in (400 − 50) × 3 = 1050 stimuli. Dividing the stimuli further into ‘low’ and ‘high’ tone-frequencies, resulted in about 525 stimuli for the analysis of each combination of neuron and test frequency. The reduced representation determines the prediction error associated with each stimulus for each state −log_2_ *P*(*future*|*m*). For each stimulus we considered the previous *N* stimuli, using them to estimate the probability of entering each state *m* by *P(m|past)*. These probabilities served as weights in calculating the regression between spike counts and prediction error values. Using the method of weighted linear regression allowed us to take the uncertainty in the unknown state *m* into account. Finally, the fraction of explained variance based on this weighted linear regression (weighted *r*^2^) was used to measure the goodness-of-fit associated with each one of the reduced representations. For each combination of neuron and test frequency we denoted the highest fraction of explained variance over the entire set of reduced representations by $r_{max}^{2}$.

We considered reduced representations that achieved a score of at least 90% of $r_{max}^{2}$ as ‘good representations’. For each combination of neuron and test frequency used in the main analysis, we constructed two-dimensional binary maps indicating the set of ‘good representations’ over the parameter space (complexity, predictive power and past duration: see **Fig 6C–6E**). For **Fig 6**, We calculated the averages of these binary maps over a subset of the population, which had high explanatory power ($r_{max}^{2}\geq 10\%$; *n* = 34/117).
